# Supplementary material for: Modeling glioblastoma heterogeneity as a dynamic network of cell states
Source: Mol Syst Biol. 2021 Sep 16;17(9):e10105. doi: 10.15252/msb.202010105 (PMC8444284; doi:10.15252/msb.202010105)
Supplement: Supplementary file 5 — Source Data for Figure 3 [file MSB-17-e10105-s001.zip › Figure3A_sourcedata/GSEA_3065/hallmarks_state4.GseaPreranked.1623416296068/HALLMARK_REACTIVE_OXYGEN_SPECIES_PATHWAY.html]

Details for gene set HALLMARK\_REACTIVE\_OXYGEN\_SPECIES\_PATHWAY[GSEA]

|  || Dataset | state4 |
| Phenotype | NoPhenotypeAvailable |
| Upregulated in class | na\_pos |
| GeneSet | HALLMARK\_REACTIVE\_OXYGEN\_SPECIES\_PATHWAY |
| Enrichment Score (ES) | 0.38887388 |
| Normalized Enrichment Score (NES) | 1.2877097 |
| Nominal p-value | 0.12777779 |
| FDR q-value | 0.19783127 |
| FWER p-Value | 0.745 |
Table: GSEA Results Summary

  

Fig 1: Enrichment plot: HALLMARK\_REACTIVE\_OXYGEN\_SPECIES\_PATHWAY      
 Profile of the Running ES Score & Positions of GeneSet Members on the Rank Ordered List

  

| PROBE | GENE SYMBOL | GENE\_TITLE | RANK IN GENE LIST | RANK METRIC SCORE | RUNNING ES | CORE ENRICHMENT || 1 | FTL |  |  | 13 | 0.466 | 0.1319 | Yes |
| 2 | LAMTOR5 |  |  | 144 | 0.225 | 0.1755 | Yes |
| 3 | GPX4 |  |  | 183 | 0.210 | 0.2297 | Yes |
| 4 | SOD1 |  |  | 396 | 0.152 | 0.2395 | Yes |
| 5 | PRDX1 |  |  | 416 | 0.149 | 0.2792 | Yes |
| 6 | JUNB |  |  | 490 | 0.139 | 0.3074 | Yes |
| 7 | ATOX1 |  |  | 572 | 0.129 | 0.3314 | Yes |
| 8 | PRDX2 |  |  | 634 | 0.122 | 0.3566 | Yes |
| 9 | NDUFB4 |  |  | 649 | 0.120 | 0.3889 | Yes |
| 10 | CAT |  |  | 1179 | 0.077 | 0.3262 | No |
| 11 | PRDX6 |  |  | 1506 | 0.062 | 0.2916 | No |
| 12 | HMOX2 |  |  | 1572 | 0.059 | 0.2982 | No |
| 13 | NDUFS2 |  |  | 1752 | 0.054 | 0.2849 | No |
| 14 | PTPA |  |  | 2370 | 0.038 | 0.1967 | No |
| 15 | PDLIM1 |  |  | 2537 | 0.034 | 0.1799 | No |
| 16 | STK25 |  |  | 2956 | 0.026 | 0.1202 | No |
| 17 | SBNO2 |  |  | 2973 | 0.025 | 0.1248 | No |
| 18 | ERCC2 |  |  | 3033 | 0.024 | 0.1224 | No |
| 19 | GCLC |  |  | 3344 | 0.017 | 0.0775 | No |
| 20 | G6PD |  |  | 3603 | 0.009 | 0.0386 | No |
| 21 | MSRA |  |  | 3739 | 0.004 | 0.0180 | No |
| 22 | NDUFA6 |  |  | 4074 | -0.014 | -0.0316 | No |
| 23 | PRNP |  |  | 4778 | -0.066 | -0.1253 | No |
| 24 | TXN |  |  | 4891 | -0.074 | -0.1219 | No |
| 25 | CDKN2D |  |  | 4933 | -0.077 | -0.1062 | No |
| 26 | GLRX2 |  |  | 5268 | -0.107 | -0.1290 | No |
| 27 | PFKP |  |  | 5471 | -0.128 | -0.1247 | No |
| 28 | NQO1 |  |  | 5566 | -0.138 | -0.1002 | No |
| 29 | TXNRD1 |  |  | 5782 | -0.179 | -0.0832 | No |
| 30 | SELENOS |  |  | 5922 | -0.213 | -0.0444 | No |
| 31 | PRDX4 |  |  | 6155 | -0.345 | 0.0173 | No |
Table: GSEA details [plain text format]

  

Fig 2: HALLMARK\_REACTIVE\_OXYGEN\_SPECIES\_PATHWAY: Random ES distribution      
 Gene set null distribution of ES for **HALLMARK\_REACTIVE\_OXYGEN\_SPECIES\_PATHWAY**
